# ﻿Two new species of *Paraphlomis* (Lamiales, Lamiaceae) from limestone karsts in Guangdong Province, China

**DOI:** 10.3897/phytokeys.243.114415

**Published:** 2024-06-27

**Authors:** Wan-Yi Zhao, Qin-Dai Xiong, Rang-Min Wu, You-Hong Zeng, Zhi-Bin Xie, Ya-Ping Chen, Qiang Fan

**Affiliations:** 1 State Key Laboratory of Biocontrol and Guangdong Provincial Key Laboratory of Plant Stress Biology, School of Life Sciences, Sun Yat-sen University, Guangzhou 510275, China Sun Yat-sen University Guangzhou China; 2 Qingyuan Forestry Bureau, Qingyuan 511518, China Qingyuan Forestry Bureau Qingyuan China; 3 Qingyuan Jinji Forestry Farm, Qingyuan 513029, China Qingyuan Jinji Forestry Farm Qingyuan China; 4 Yingde Forestry Bureau, Qingyuan 513099, China Yingde Forestry Bureau Qingyuan China; 5 CAS Key Laboratory for Plant Diversity and Biogeography of East Asia, Kunming Institute of Botany, Chinese Academy of Sciences, Kunming 650201, China Kunming Institute of Botany, Chinese Academy of Sciences Kunming China

**Keywords:** IUCN, limestone, new taxon, Paraphlomideae, phylogeny, Qingyuan, RAD-seq

## Abstract

*Paraphlomisqingyuanensis* and *P.baiwanensis* (Lamiaceae), two new species from the limestone area in Guangdong Province, China, are described. Morphologically, both species belong to P.ser.Subcoriaceae C.Y. Wu & H.W. Li. A close relationship between the two new and *P.subcoriacea* was revealed by molecular phylogenetic analyses based on ETS and ITS. Further morphological and population genetic evidence indicated that they are distinct species in *Paraphlomis*. According to the IUCN Red List Categories and Criteria, *P.qingyuanensis* and *P.baiwanensis* were assessed as Endangered (EN) and Deficient (DD), respectively.

## ﻿Introduction

*Paraphlomis* (Prain) Prain is a member of the tribe Paraphlomideae Bendiksby (Lamiaceae, Lamioideae) (Bendiksby et al. 2011; Li et al. 2016; Zhao et al. 2021). Most species of *Paraphlomis* are distributed in southern China, with several species occurring in the Himalayas, Korea and Southeast Asia (Li and Hedge 1994; Wu and Li 1997; Ko et al. 2014; Chen et al. 2021). Previous molecular phylogenetic studies of *Paraphlomis* revealed that the genus was not monophyletic, because species of *Matsumurella* were recovered within it (Chen et al. 2021; Chen et al. 2022b; Guo et al. 2023). In general, *Paraphlomis* is characterized by its herbaceous habit, actinomorphic calyx with five lobes less than half as long as the tube, corolla 2-lipped (1/3) with hairy upper lip but hardly bearded along the margin, included stamens and an apically truncate ovary (Wu and Li 1977; Bendiksby et al. 2011; Ko et al. 2014; Chen et al. 2021).

As currently circumscribed, a total of 37 species and seven varieties are recognised within *Paraphlomis* (Li and Hedge 1994; Chen et al. 2022a, 2022b; Yuan et al. 2022; Guo et al. 2023; Yan et al. 2023). China is the distribution center of *Paraphlomis*, with 23 species documented in the Flora of China (Li and Hedge 1994). In recent years, a number of new species and infraspecies of *Paraphlomis* were reported in China, including P.javanicavar.pteropoda D. Fang & K.J. Yan and P.javanicavar.angustifoliaf.albinervia D. Fang & K.J. Yan (Yan and Fang 2009); *P.breviflora* B.Y. Ding, Y.L. Xu & Z.H. Chen (Ding et al. 2019); *P.kuankuoshuiensis* R.B. Zhang, D. Tan & C.B. Ma (Zhang et al. 2020); *P.jiangyongensis* X.L. Yu & A. Liu and *P.coronata* (Vaniot) Y.P. Chen & C.L. Xiang (Chen et al. 2021); *P.nana* Y.P. Chen, C. Xiong & C.L. Xiang (Chen et al. 2022c); *P.longicalyx* Y.P. Chen & C.L. Xiang (Chen et al. 2022a); *P.hsiwenii* Y.P.Chen & XiongLi (Chen et al. 2022b); *P.strictiflora* J.C.Yuan, B.Chen & C.L.Xiang (Yuan et al. 2022); *P.jinggangshanensis* Boufford, W.B. Liao & W.Y. Zhao (Zhao et al. 2022); *P.yingdeensis* W.Y.Zhao, Y.Q.Li & Q.Fan (Guo et al. 2023), *P.caloneura* K.J.Yan, Y.P.Chen & Y.Feng Huang (Yan et al. 2023).

During a botanical expedition of the limestone area in Qingyuan city, Guangdong Province in 2023, we discovered two unknown species of *Paraphlomis*. The calyx teeth of the two unknown species extended into wings from veins, which are consistent with the characteristics of Paraphlomisser.Subcoriaceae C.Y. Wu et H.W. Li (Li 1965; Wu and Li 1977). However, their morphological characters differ from the two known two species of the series, *P.subcoriacea* C. Y. Wu ex H. W. Li and *P.brevifolia* C. Y. Wu & H. W. Li. Thus, we suspected both of them were undescribed species. After careful field observations, morphological comparisons with other species of *Paraphlomis*, and molecular phylogenetic studies, we confirmed that they were new species and named them as *P.qingyuanensis* W.Y. Zhao, R.M. Wu & Q. Fan and *P.baiwanensis* W.Y. Zhao, Y.P. Chen & Q. Fan.

## ﻿Materials and methods

### ﻿Morphological study

The flowering and fruiting plants of the two new species were examined in the field from August to December in 2023 and compared with herbarium specimens deposited in IBSC, KUN, GCMI and SYS (herbarium acronyms follow Thiers 2023). The two putative new species were most similar to *Paraphlomissubcoriacea* and *P.brevifolia*. We have carried out several field work trips to the collection site of the type specimens of these two species from December 2023 to April 2024. Unfortunately, we did not find *P.brevifolia* in the field due to a lack of detailed collection site information (Li 1965). Therefore, the morphological features contrasting with those of *P.brevifolia* were based on its type specimens (IBSC0005124). All morphological characteristics were measured using dissecting microscopes.

### ﻿Phylogenetic analyses

The nuclear ribosomal internal and external transcribed spacers (ITS and ETS) were used for reconstructing the phylogeny of the suspected new species and related taxa based on previous study (Chen et al. 2021; Zhao et al. 2022). Most sequences were downloaded from GenBank, except for the two nuclear ribosomal DNA (nrDNA) sequences of the two new species and *Paraphlomissubcoriacea*, which were newly sequenced in the present study. Genomic DNA of the suspected new species was extracted from silica-gel-dried leaves using the modified 2× CTAB procedure of Doyle and Doyle (1987). The ITS and ETS sequences were amplified with primer pairs 17SE/26SE (Sun et al. 1994) and ETSB/IGS (Beardsley and Olmstead 2002), respectively, with PCR amplification and sequencing following Chen et al. (2016). A total of 49 accessions representing 30 species and four varieties/subspecies of *Paraphlomis* and two species of *Matsumurella* were sampled in the phylogenetic study. *Phlomoidesbracteosa* (Royle ex Benth.) Kamelin & Makhm. and *Phlomisfruticosa* Sieber ex C. Presl were selected as the outgroups. The GenBank accession numbers are listed in Appendix 1.

Nucleotide sequences were aligned using MAFFT 7 (Katoh and Standley 2013). After removing aligned columns with more than 70% missing data using Phyx (Brown et al. 2017), the two nrDNA regions were concatenated for phylogenetic reconstruction. The phylogenetic relationships were assessed using the Bayesian inference (BI) and maximum likelihood (ML) methods, and both were implemented on the online server Cyberinfrastructure for Phylogenetic Research Science (CIPRES) Gateway (http://www.phylo.org/; Miller et al. 2010). The BI analysis was carried out using MrBayes 3.2.7a (Ronquist et al. 2012) and the ML analysis using RAxML-HPC2 (Stamatakis 2014), with detailed settings following that of Chen et al. (2021). The resulting BI tree with posterior probabilities (PP) and best-scoring ML tree with bootstrap support (BS) values were visualized and annotated using TreeGraph 2 (Stover and Müller 2010).

### ﻿Population genetic structure analyses

To investigate the phylogenetic relationship between the two newly discovered species and their close relatives, we further conducted an analysis of their population genetic structure. A total of 84 individuals were sampled from seven populations of the two putative *Paraphlomis* new species and its close relative *P.subcoriacea* (Appendix 2). The fresh leaves were dried and stored with silica gel, and then sent to JieRui BioScience Co. Ltd. (Guangzhou, China) for DNA extraction, ddRAD-seq library preparation, and Illumina sequencing. The produced raw data was processed with the software Stacks 2.55 (Catchen et al. 2013). The procedure “process_radtags” was used to demultiplex RAD tags, “denovo_map.pl” to process all the fastq files, and “populations” to filter the data by setting “--min-maf 0.05 --max-obs-het 0.7 -R 0.8 –write-random-snp –vcf”. The produced vcf file was transformed to ped file using bed file using software vcftools 0.1.16 (Danecek et al. 2011), and to bed file using the software Plink v1.90 (Chang et al. 2015). The produced bed file was used to perform Bayesian cluster analysis with the software ADMIXTURE v1.3.0 (Alexander et al. 2009), in which the number of groups (K) was set from 1–6 and the optimal K was determined by the minimum value of cross-validation error (CV). Principal coordinate analysis (PCA) was performed with Plink, and python script were used to draw the scatter diagram.

## ﻿Results and discussion

**Figure 1.**
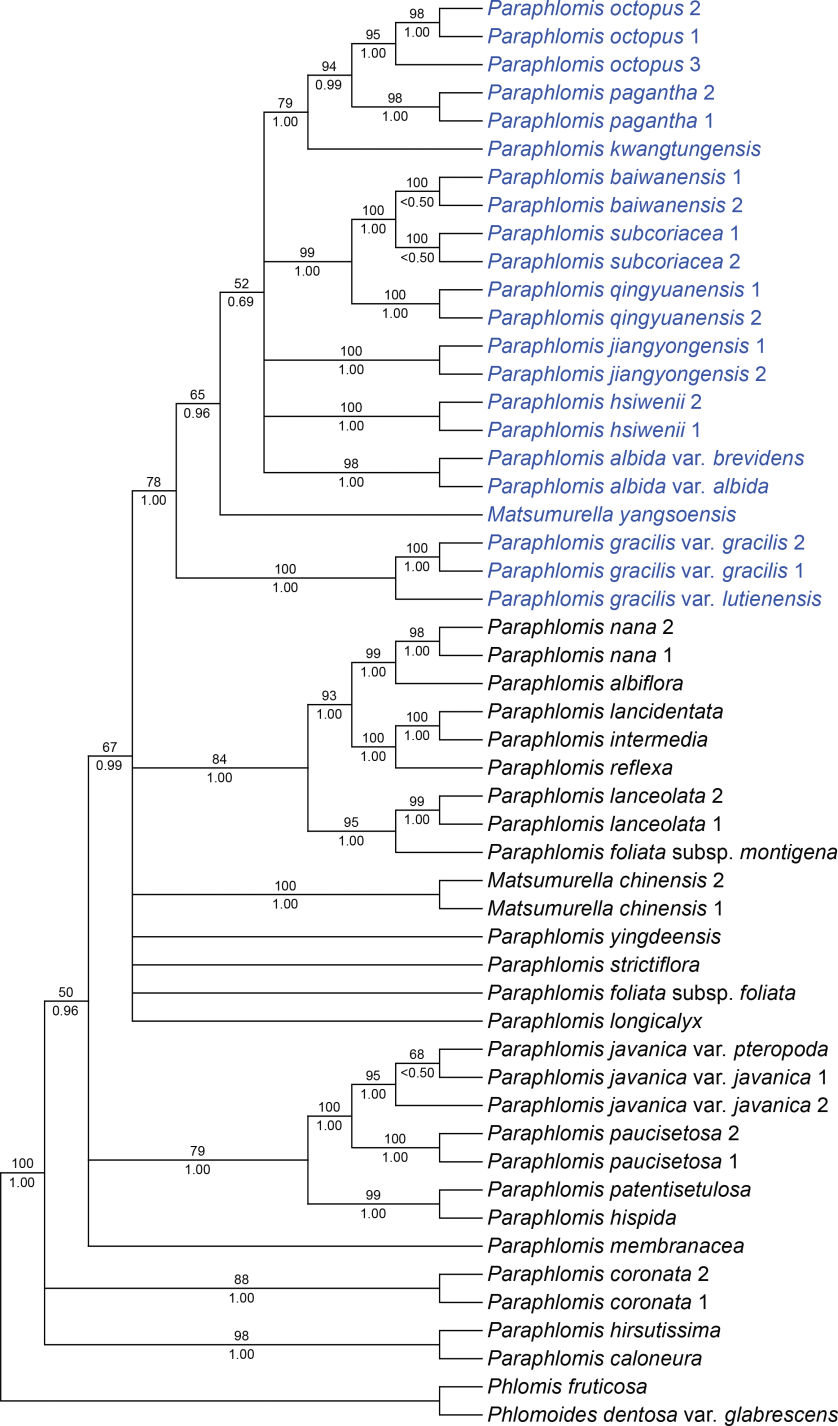
The best-scoring maximum likelihood tree of *Paraphlomis* inferred from concatenated nrDNA (ETS and ITS) dataset. Support values ≥ 50% BS or 0.50 PP are displayed above and below the branches, respectively. Multiple accessions of the same species are numbered according to Appendix 1.

**Figure 2.**
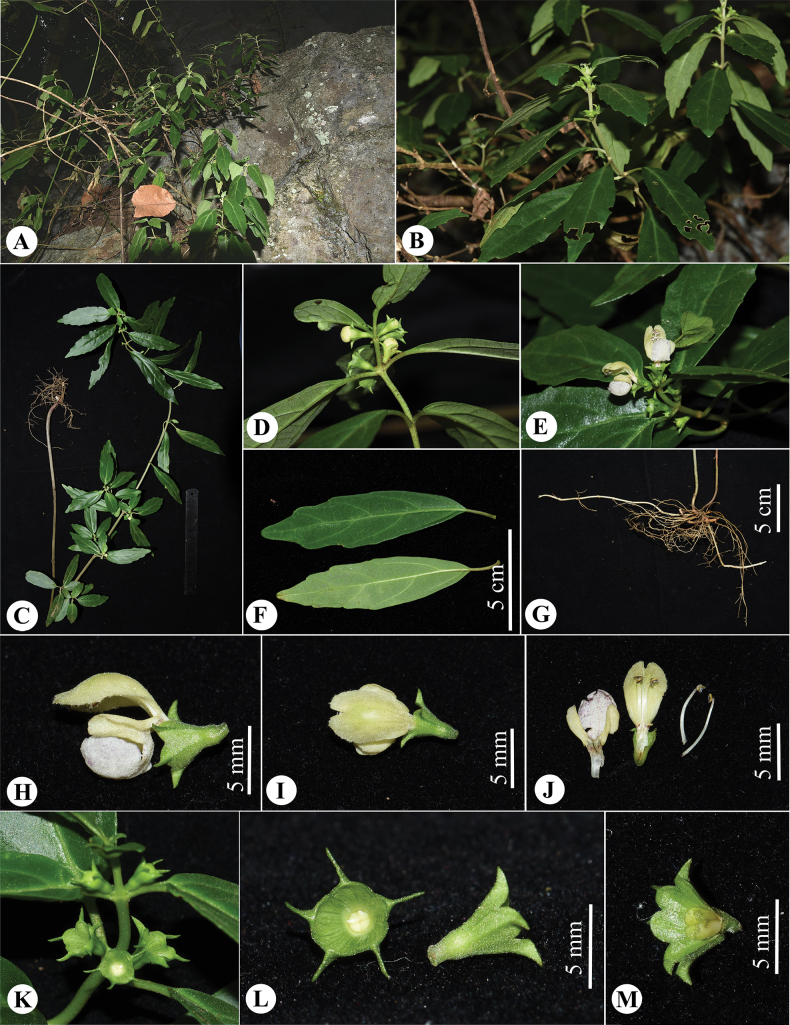
*Paraphlomisqingyuanensis***A** habitat **B** plants **C** individual **D** inflorescences with unopened flowers **E** opening flowers **F** leaves **G** stolons and roots **H** lateral view of flower **I** upper lip of flower **J** dissected corolla and stamens **K** infructescence **L** frontal and lateral view of calyx **M** dissected calyx and seeds. (Photographs: **A, B** by Qin-Dai Xiong; **C–G** by Wan-Yi Zhao).

The combined nuclear data set was 1211 bp (773 bp for ITS, 438 bp for ETS) in length, including 347 variable sites (165 for ITS, 182 for ETS) and 200 parsimony-informative characters (87 for ITS, 113 for ETS). The resulting phylogenetic tree of *Paraphlomis* in this study was similar to that in previous studies (Chen et al. 2021; Zhao et al. 2022). The two accessions of *P.baiwanensis* grouped together (Fig. 1: BS = 100%/PP < 0.50) and this new species was sister to *P.subcoriacea* (Fig. 1: BS = 100%/PP = 1.00). The *P.baiwanensis*-*P.subcoriacea* clade was further sister to another new species, *P.qingyuanensis* (Fig. 1: BS = 99%/PP = 1.00). All three species were nested within the previously suggested “Clade III” by Chen et al. (2021) (Fig. 1: BS = 78%/PP = 1.00). Species of “Clade III” (marked in blue in Fig. 1) are characterized by hairy nutlets/ovaries (Chen et al. 2021; 2022b). The nutlets of both new species are sparsely hispid and densely glandular at apex (Figs 2M, 5I, 6H), which confirmed the significance of nutlet morphology for the infrageneric classification of *Paraphlomis* (Chen et al. 2021).

The close relationships among the two new species and *P.subcoriacea* are also supported by morphological evidence. In morphology, the two putative new species are most similar to *Paraphlomisbrevifolia* C. Y. Wu et H. W. Li and *P.subcoriacea* C. Y. Wu ex H. W. Li. They share the features such as conspicuously extended calyx teeth, and the fact that they are also growing in a limestone habitat. A comparison of their morphological features is presented in Table 1 and Fig. 4.

**Table 1.** Morphological comparisons among *Paraphlomisbaiwanensis*, *P.brevifolia*, *P.qingyuanensis*, and *P.subcoriacea*.

Characters	*P.qingyuanensis*	*P.baiwanensis*	*P.subcoriacea*	*P.brevifolia*
**Stem**	50–90 cm, slender, 1.4–3.1 mm in diam	50–80 cm tall, erect, stout, 3.6–5.3 mm in diam	ca. 60 cm, ca. 3.6 mm in diam	ca. 40 cm, ca. 2.5 mm in diam
**Stem branching**	much branched	unbranched or 2–3 branched	unbranched or 2–3 branched	unbranched
**Length of petiole**	0.4–1.6 cm	0.6–1.3 cm	1.0–1.3 cm	0.3–0.6 cm
**Leaf size**	2.7–9.5 cm long, 1.2–2.5 cm wide	12.5–18.7 cm long, 2.6–4.8 cm wide	7–15 cm long, 1.5–3.2 cm wide	5–8 cm long, 2.4–3.4 cm wide
**Leaf base**	cuneate, not decurrent	cuneate or abruptly obtuse, not decurrent	attenuate or abruptly obtuse, not decurrent	obtuse to rounded, not decurrent
**Leaf texture**	papery	leathery	thin leathery	thin leathery
**Leaf vein**	2–4 (-5) pairs	5–7 (-8) pairs	5–6 pairs	4–5 pairs
**Calyx teeth**	subtruncate, extended into wings from veins	subtruncate, extended into wings from veins	subtruncate, extended into wings from veins	triangular, conspicuously extended into wings from veins
**Corolla**	upper lip yellow, lower lip red with purple spots	white	white or purple-white	unknown

The putative new species *Paraphlomisqingyuanensis* differs from *P.brevifolia* by its slender and much branched stem (vs. unbranched) (Figs 2C, 4A), leaves without glandular (vs. abaxially golden glandular) (Table 1). Furthermore, the reticulate veins and pilose indumentum on leaves of *P.brevifolia* are more obvious (Fig. 4B). *P.qingyuanensis* could also be easily distinguished from *P.subcoriacea* by its papery leaves (vs. thin leathery), fewer leaf veins, 2–4 pairs (vs. 5–6 pairs), and smaller leaf size (Table 1, Fig. 4A, D).

*Paraphlomisbaiwanensis* is most similar to *P.subcoriacea*. They share such features as leaves lanceolate, 5–7 lateral vein pairs, and leaf base shape (Table 1). However, *P.baiwanensis* could be easily distinguished from the latter by its stout stem (diam. 3.6–5.3 mm vs. ca. 3.6 mm), leathery leaves (vs. thin leathery), and larger leaves size (12.5–18.7 cm × 2.6–4.8 cm vs. 7–15 cm × 1.5–3.2 cm) (Table 1, Fig. 4C, D). Furthermore, the distribution areas of the two species are separated by a distance of approximately 45 km, exhibiting complete non-overlap (Fig. 7A). The results of population genetic analysis also confirmed significant differences in their genetic structure (Fig. 7B).

Bayesian cluster analysis showed lowest CV value as *K* = 3, each of the three species *P.baiwanensis* (P1), *P.subcoriacea* (P2–4), and *P.qingyuanensis* (P5–7) possesses a unique gene pool, and no gene admixture is observed in any individuals (Fig. 7B). PCA analysis reveals similar results in which the 84 individuals are assigned into three groups and individuals of the same species are clustered into the same group (Fig. 7C). These results showed that individuals of the three species can be separated clearly from each other based on genomic data, strongly supported their species status and no obvious gene flow was observed among the three species.

### ﻿Taxonomic treatment

#### ﻿ Paraphlomis qingyuanensis

Taxon classificationPlantaeLamialesLamiaceae

W.Y.Zhao, R.M.Wu & Q.Fan sp. nov.

972DB679-0048-5138-81CE-40A3870F8F9B

urn:lsid:ipni.org:names:77344362-1

**Figure 3.**
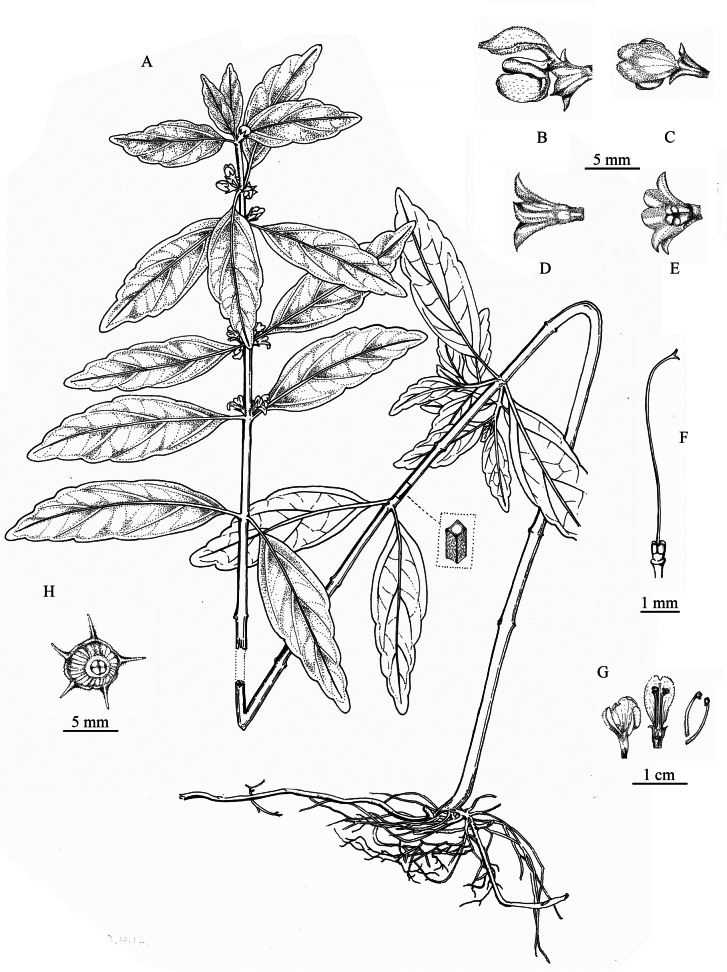
Line drawing of *Paraphlomisqingyuanensis***A** plant **B** lateral view of flower **C** upper lip of flower **D** lateral view of calyx **E** inner view of calyx **F** style **G** dissected corolla and stamens **H** front view of calyx (Drawn by Zhong-Jin Wang).

**Figure 4.**
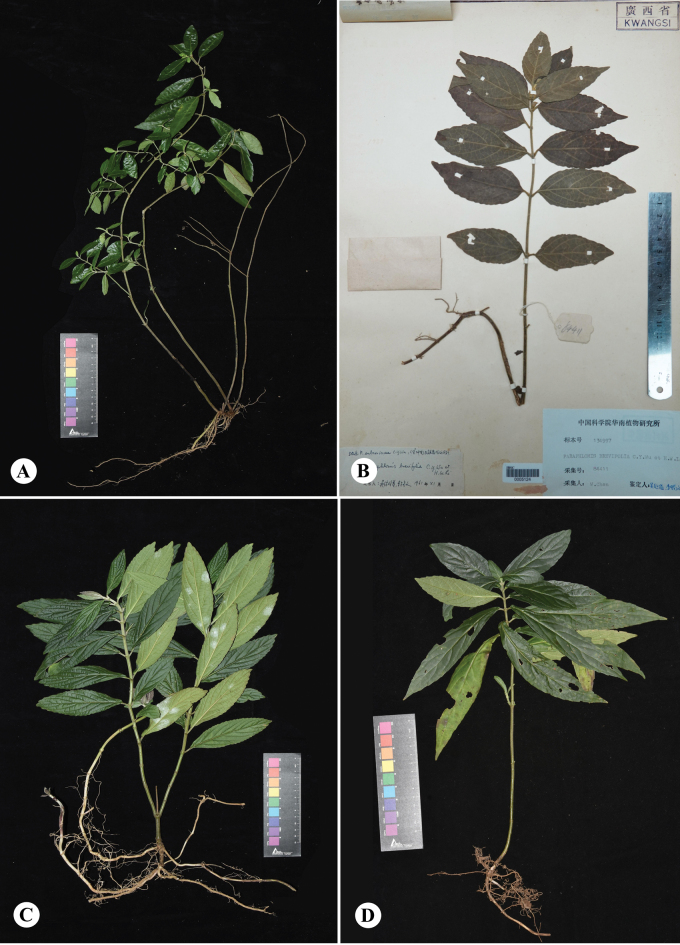
Morphological comparison of *Paraphlomisqingyuanensis*, *P.brevifolia*, *P.baiwanensis*, and *P.subcoriacea*. *Paraphlomisqingyuanensis***A** plant of *P.qingyuanensis***B** plant of *P.brevifolia***C** plant of *P.baiwanensis***D** plant of *P.subcoriacea* (Photographs by Wan-Yi Zhao; photographs of *P.brevifolia* from isotype *W. Chen 84411*, IBSC0005124).

Figs 2 , 3 , 4A 清远假糙苏

##### Type.

China. Guangdong Province: Yingde City, Huanghua Town, near Hegushi, on the limestone valley, 24°13'N, 112°56'E, alt. 135 m, 7 September 2023, *Xiong Qin-Dai ZWY-3793* (holotype: SYS00236954! isotypes: KUN, SYS00236955!, SYS00236968!, SYS00236969!).

##### Diagnosis.

*Paraphlomisqingyuanensis* is morphologically similar to *P.brevifolia* and *P.subcoriacea*, but differs from the latter two species by its slender and much branched stem, papery leaves, inconspicuous reticulate veining, and fewer leaf veins.

##### Description.

***Herbs*** perennial, 50–90 cm tall; stoloniferous, with sparsely villous. **Stems** erect or decumbent, slender and much branched, 4-angled, diameter 1.4–3.1 mm, densely retrorse pilose. ***Leaves*** opposite; lamina ovate to oblong, papery, 2.7–9.5 cm long, 1.2–2.5 cm wide, apex obtuse, base cuneate, margin crenate-serrate; adaxially green, abaxially light green, sparsely pilose on both sides, more densely on viens; lateral veins 2–4 (-5)-paired; petiole 0.4–1.6 cm long, densely pilose. ***Verticillasters*** born in upper leaf axils, cymes 1–4-flowered; bracteoles inconspicuous, ca. 0.5 mm long, early deciduous; pedicels ca. 1 mm long, sparsely pilose. ***Calyx*** light green, obconical, 4.5–5 mm long, outside with sparsely pilose and white glandular, glabrous inside, conspicuously 10-veined; teeth 5, inconspicuous so that calyx mouth appears subtruncate, extended into wings from veins as 1.5–2.0 mm long. ***Corolla*** 1.0–1.2 cm long; tube ca. 0.3 cm long, ca. 1 mm in diam., straight, inside of throat dark purple, with pubescent annulate; 2-lipped, villous outside, upper lip oblong, erect and galeate, apex undulate or bilobate, ca. 8 mm long, ca. 4 mm wide; lower lip reflexed, ca. 4.5 mm long, 3-lobed, medium lobe largest, suborbicular, ca. 5 mm long, ca. 4.5 mm wide, apex emarginate, white, dotted with purplish-red spots, lateral lobes ovate, ca. 3.5 mm long, ca. 3 mm wide, apex obtuse, yellow. ***Stamens*** 4, inserted above middle and upper of corolla tube, straight, included, filaments flat, sparsely puberulent-villous, anther cells 2, ovoid, glabrous. ***Style*** filiform, included, glabrous, apex subequally 2-lobed, ca. 1 cm long. ***Ovary*** 4-loculed, truncate at apex. ***Nutlets*** triquetrous-oblong, ca. 2.5 mm long, apex sparsely hispid and glandular.

##### Distribution, habitat and conservation status.

Currently, only three populations of *Paraphlomisqingyuanensis* were found in Huanghua Town of Yingde City, Qingyuan City in Guangdong Province (Fig. 7A). These populations were located in the subtropical monsoon climate region, in a large area of karst landform. The distribution area of *P.qingyuanensis* is extremely fragmented, and it is not within a protected area. Human activity, such as forestry production and tourism, have a negative effect on population regeneration. Thus, *P.qingyuanensis* is here suggested to be endangered (EN) according to IUCN categories guidelines B2(a, b(iii)) (AOO < 500 km^2^, number of locations <5, and habitat affected by human activities) (IUCN Standards and Petitions Subcommittee 2022).

##### Phenology.

Flowering was observed from August to December, and fruiting from September to December.

##### Etymology.

The specific epithet “*qingyuanensis*” is derived from the type locality of the new species, i.e. Qingyaun City in Guangdong Province, China.

##### Additional specimens examined.

*Paraphlomisqingyuanensis* (paratypes): China. Guangdong Province: Qingyuan City, Yingde City, Huanghua Town, near Hegushi, on the limestone valley, 24°13'N, 112°56'E, alt. 132 m, 6 September 2023, *Qin-Dai Xiong QYK-HH-156* (SYS); Yingde city, Huanghua town, Hegushi, 24°13'35.7"N, 112°56'08.97"E, alt. 114 m, 16 December 2023, *Qiang Fan 20255* (SYS); Yingde city, Huanghua town, near Hegushi, 24°13'22.71"N, 112°56'05.71"E, alt. 159 m, 26 December 2023, *Qiang Fan & Qin-Dai Xiong QYK-HH-1904* (SYS); Yingde city, Huanghua town, Huanghua park, 24°12'03.46"N, 112°54'10.05"E, alt. 205 m, 25 December 2023, *Qiang Fan & Qin-Dai Xiong QYK-HH-1882* (SYS).

#### ﻿ Paraphlomis baiwanensis

Taxon classificationPlantaeLamialesLamiaceae

W.Y.Zhao, Y.P.Chen & Q.Fan sp. nov.

6A6801CD-587F-52BB-93A7-284B893883D7

urn:lsid:ipni.org:names:77344363-1

**Figure 5.**
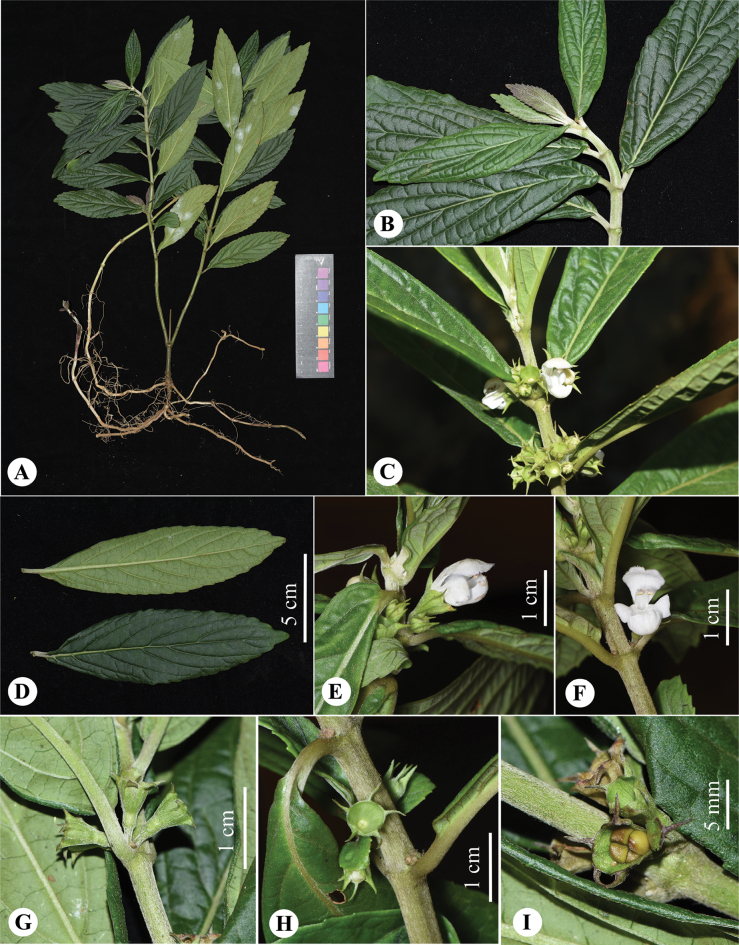
*Paraphlomisbaiwanensis***A** plant **B** young branch and leaves **C** flowering branch **D** leaves **E** lateral view of flower **F** front view of flower **G** lateral view of calyx **H** front view of calyx **I** seeds (Photographs: **A, B, D, G, I** by Wan-Yi Zhao; **C** by Qin-Dai Xiong; **E-F, H** by Qiang Fan).

**Figure 6.**
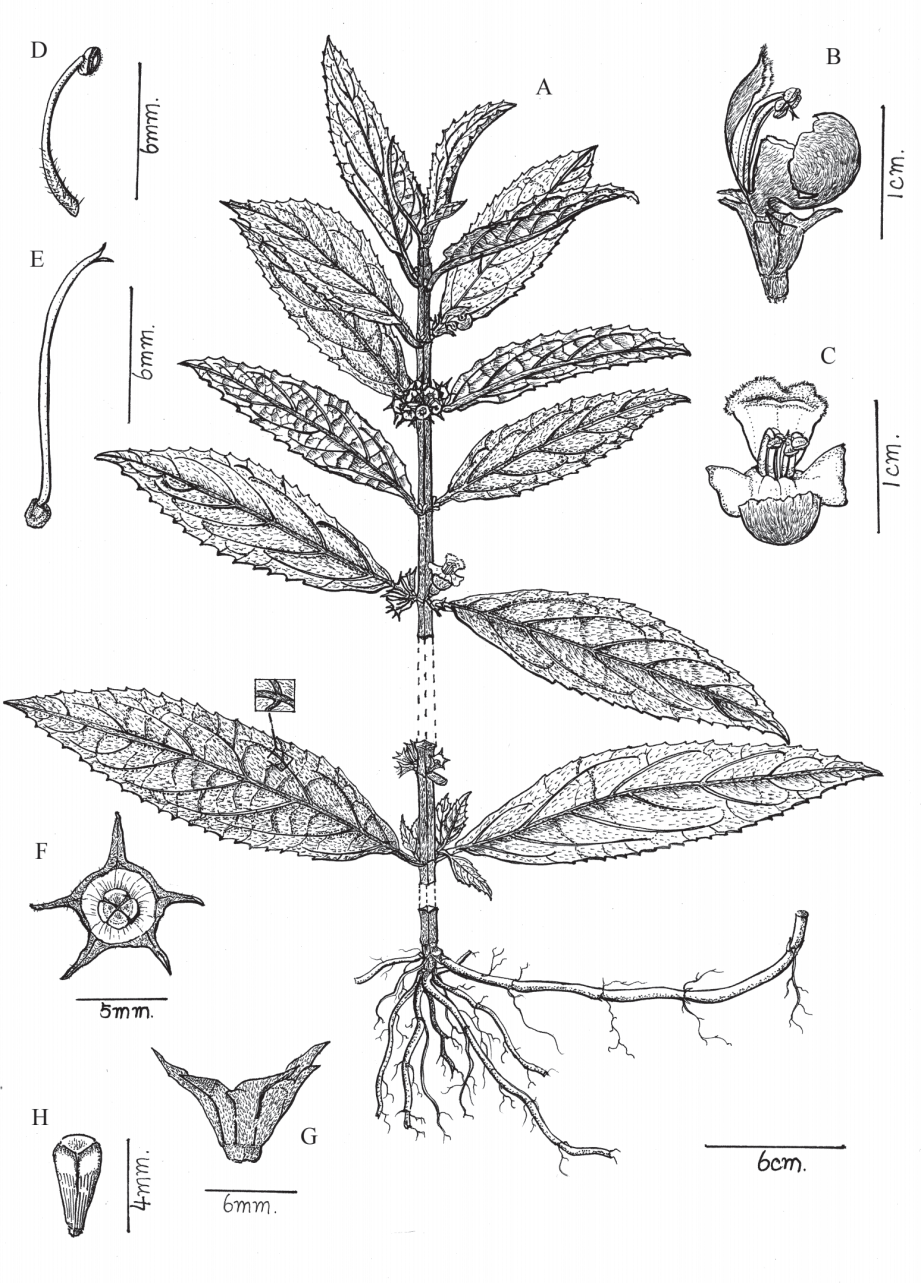
Line drawing of *Paraphlomisbaiwanensis***A** plant **B** lateral view of a flower **C** frontal view of corolla **D** stamen **E** style **F** front view of calyx **G** lateral view of calyx tube **H** seed (Drawn by Rong-En Wu).

Figs 4C , 5 , 6 白湾假糙苏

##### Type.

China. Guangdong Province: Qingyuan city, Qingxin district, Baiwan town, Hecang village, on the limestone cliff, 24°15'09.40"N, 112°46'47.32"E, alt. 310 m, 15 December 2023, *Qiang Fan 20251* (holotype: SYS00236952!; isotypes: KUN, SYS00236953!).

##### Diagnosis.

*Paraphlomisbaiwanensis* is morphologically similar to *P.subcoriacea*, but differs from the latter by its stout stem, larger and leathery leaves, and larger flower.

##### Description.

***Herbs*** perennial, 50–80 cm tall; stoloniferous, with villous indumentum. ***Stems*** erect, stout, unbranched or 2–3 branched, 4-angled, diameter 3.6–5.3 mm, with densely retrorse pilose hairs. ***Leaves*** opposite; lamina long ovate to lanceolate, leathery, 12.5–18.7 cm long, 2.6–4.8 cm wide, apex acuminate, base cuneate or abruptly obtuse, margin serrulate; adaxially dark green, with densely pilose, abaxially light green with densely brown glandular, sparsely pilose, more densely on veins; lateral veins 5–7 (-8)-paired, raised abaxially and deeply impressed adaxially, anastomosing at leaf margin; petiole 0.6–1.3 cm long, densely pilose. ***Verticillasters*** borne in upper leaf axils, cymes (2-) 5–9-flowered; bracteoles inconspicuous, early deciduous; pedicels ca. 1.2–2.0 mm long, densely pilose. ***Calyx*** light green, obconical, 5.2–5.7 mm long, glabrous inside, outside with densely retrorse pilose, conspicuously 10-veined; calyx teeth 5, inconspicuous, throat appearing subtruncate, calyx veins extended into wings as 2.5–3.0 mm long. ***Corolla*** 1.5–2.1 cm long, white; tube ca. 7 mm long, ca. 2 mm in diam., straight, inside of throat with pubescent annulate; 2-lipped, villous outside, upper lip oblong, galeate, apex undulate, ca. 8–10 mm long, ca. 3.8–5.5 mm wide; lower lip reflexed, ca. 7 mm long, 3-lobed, medium lobe largest, suborbicular, ca. 6.5 mm long, ca. 6 mm wide, apex emarginate, lateral lobes ovate, ca. 4.5 mm long, ca. 3.2 mm wide, apex obtuse. ***Stamens*** 4, inserted above middle and upper of corolla tube, straight, included, filaments flat, 7–9 mm long, sparsely puberulent-villous, anther cells 2, ovoid, glabrous. ***Style*** filiform, included, glabrous, apex subequally 2-lobed, ca. 1 cm long. ***Ovary*** 4-loculed, truncate at apex. ***Nutlets*** triquetrous-oblong, ca. 3.8 mm long, apex sparsely hispid.

##### Distribution, habitat and conservation status.

*Paraphlomisbaiwanensis* is currently known to occur only in Baiwan town, Guangdong in one population numbering less than one hundred individuals. It was observed to grow on limestone cliffs at altitudes about 300 m. Its known population was located in Qingxin Baiwan Provincial Nature Reserve of Guangdong which is well-protected. More field investigations are needed to determine its wild distribution. Therefore, the Protection level of *Paraphlomisbaiwanensis* was suggested as Data Deficient (DD) based on the IUCN Red List Criteria (IUCN Standards and Petitions Subcommittee 2022).

##### Phenology.

Flowering was observed from June to September, and fruiting from August to December.

##### Etymology.

The specific epithet “*baiwanensis*” is derived from the type locality of the new species, i.e. Qingxin Baiwan Provincial Nature Reserve of Guangdong, Qingyuan, China.

**Figure 7.**
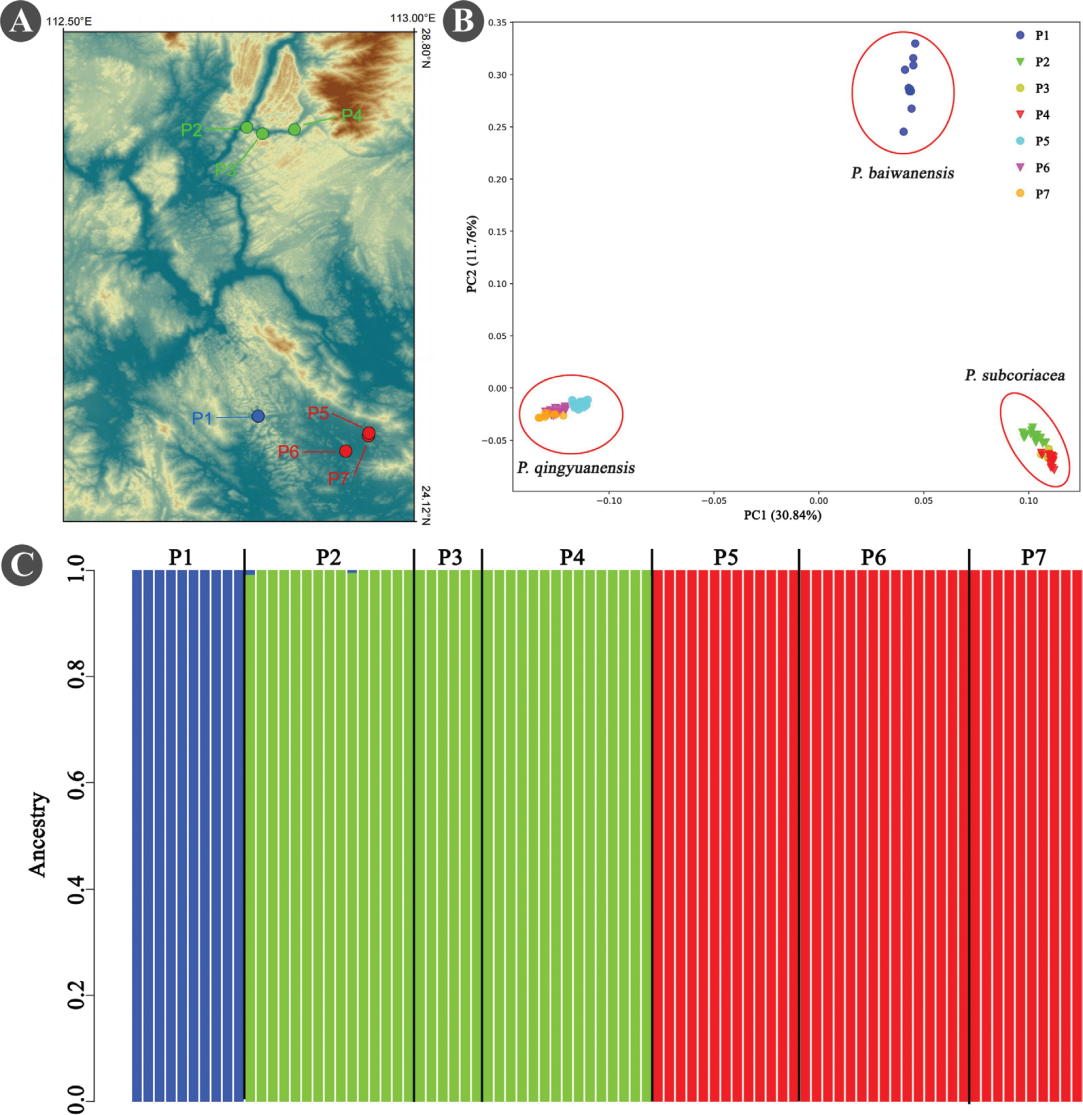
Geographic distribution and population genetic structure of *Paraphlomisbaiwanensis* (P1), *P.subcoriacea* (P2–4), and *P.qingyuanensis* (P5–7) based on Admixture analysis **A** geographic distribution of the seven populations used for analysis **B** two-dimensional clustering of genetic variation from seven populations obtained via principal component analysis **C** Populations genetic structure result based on Admixture analysis.

##### Additional specimens examined.

*Paraphlomisbaiwanensis* (paratypes): China. Guangdong Province: Qingyuan city, Qingxin district, Baiwan town, Hecang village, on the limestone cliff, 24°15'09.40"N, 112°46'47.32"E, alt. 310 m, 23 August 2023,*Yi-Wen Liu QYK-BW-1470* (SYS).

*Paraphlomisbrevifolia*: China. Guangxi Province: Luocheng City, 1939, *Wen Chen 84411* (IBSC0005124, isotype).

*Paraphlomissubcoriacea*: China. Guangdong Province: Qingyuan City, Yangshan City, Chengjia country, Wuyuan village, Baizhushan, 20 June 1956, *Liang Deng 1572* (IBSC0005130, isotype); Yangshan City, Dalang Town, Chakeng village, 7 August 1936, *Liang Deng 263* (IBK00059958; IBSC0585118); Yangshan City, Lingbei Town, Xiatianxia, 24°39'48.99"N, 112°45'41.73"E, alt. 295 m, 21 December 2023, *Qiang Fan 20257* (SYS); Yangshan City, Lingbei Town, Yanzidong, 24°39'16.92"N, 112°46'59.95"E, alt. 156 m, 27 December 2023, *Qiang Fan & Qin-Dai Xiong QYK-LB-1920* (SYS); Yangshan City, Chengjia Town, Baizhuling (collection site of type specimen), 24°39'39.56"N, 112°49'45.69"E, alt. 387 m, 27 December 2023, *Qiang Fan & Qin-Dai Xiong QYK-CJ-1911* (SYS).

## Supplementary Material

XML Treatment for Paraphlomis qingyuanensis

XML Treatment for Paraphlomis baiwanensis

## ﻿Additional information

### Author ORCIDs

Wan-Yi Zhao https://orcid.org/0000-0002-6103-1236

Qin-Dai Xiong https://orcid.org/0009-0006-3645-7362

Rang-Min Wu https://orcid.org/0009-0004-3545-1933

You-Hong Zeng https://orcid.org/0009-0004-1171-5762

Zhi-Bin Xie https://orcid.org/0009-0004-9382-8073

Ya-Ping Chen https://orcid.org/0000-0002-7502-1848

Qiang Fan https://orcid.org/0000-0003-4254-6936

### Data availability

The newly-obtained ITS and ETS sequences have been submitted to the NCBI (accession number: PP897029–PP897034; PP897950–PP897955). RAD raw data was deposited to the NCBI SRA database with BioProject PRJNA1107389. All of the other data that support the findings of this study are available in the main text.

## ﻿Appendix 1

**Table A1.** Sequence information for all samples used in present study. A “/” indicates a missing sequence. Herbarium abbreviations are listed after the vouchers. The accession numbers marked in bold represent sequences newly generated.

Taxon	Voucher	Country	ITS	ETS
*Matsumurellachinensis* (Benth.) Bendiksby 1	Y. Yang OYY00316 (KUN)	Pingxiang, Jiangxi, China	MW602147	MW602117
*Matsumurellachinensis* (Benth.) Bendiksby 2	Y. Yang OYY00131 (KUN)	Guilin, Guangxi, China	MW602148	MW602118
*Matsumurellayangsoensis* (Y.Z. Sun) Bendiksby	L. Wu & W.B. Xu 10965 (IBK)	Yangshuo, Guangxi, China	MW602142	MW602112
ParaphlomisalbidaHand.-Mazz.var.albida	A. Liu et al. LK0841 (CSFI)	Ningyuan, Hunan, China	MW602124	MW602091
Paraphlomisalbidavar.brevidens Hand.-Mazz.	Y.P. Chen EM312 (KUN)	Hezhou, Guangxi, China	MW602130	MW602098
*Paraphlomisalbiflora* (Hemsl.) Hand.-Mazz.	C.M. Tan et al. 1806393 (JJF)	Jiujiang, Jiangxi, China	/	MW602101
***Paraphlomisbaiwanensis* 1**	**Y.S. Chen et al. QY20230302 (IBSC)**	**Qingyuan, Guangdong, China**	**PP897029**	**PP897950**
***Paraphlomisbaiwanensis* 2**	**Q. Fan et al. 20251 (SYS)**	**Qingyuan, Guangdong, China**	**PP897030**	**PP897951**
*Paraphlomiscaloneura* K.J. Yan, Y.P. Chen & Y.F. Huang	W.H. Wu et al. LHT1841 (KUN)	Napo, Guangxi, China	OQ627454	OQ628080
*Paraphlomiscoronata* (Vaniot) Y.P. Chen & C.L. Xiang 1	E.D. Liu et al. 3043 (KUN)	Emeishan, Sichuan, China	MW602137	MW602107
*Paraphlomiscoronata* (Vaniot) Y.P. Chen & C.L. Xiang 2	C.L. Xiang 358 (KUN)	Jiangkou, Guizhou, China	MW602123	MW602090
Paraphlomisfoliata(Dunn)C.Y. Wu & H.W. Lisubsp.foliata	S.P. Chen s.n. (KUN)	Jiangle, Fujian, China	/	MW602097
Paraphlomisfoliatasubsp.montigena X.H. Guo & S.B. Zhou	Y.C. Dai s.n. (KUN)	Hangzhou, Zhejiang, China	OM836064	OM884453
*Paraphlomisgracilis* (Hemsl.) Kudô var. gracilis 1	A. Liu LK0931 (CSFI)	Changsha, Hunan, China	MW602134	MW602104
*Paraphlomisgracilis* (Hemsl.) Kudô var. *gracilis 2*	C.L. Xiang XCL1315 (KUN)	Chongqing, China	MW602141	MW602111
Paraphlomisgracilisvar.lutienensis (Y.Z. Sun) C.Y. Wu	C.L. Xiang XCL881 (KUN)	Shibing, Guizhou, China	MW602131	MW602099
*Paraphlomishirsutissima* C.Y. Wu & H.W. Li	Zhao & G. Chen XCL2115 (KUN)	Malipo, Yunnan, China	OQ627453	OQ628079
*Paraphlomishispida* C.Y. Wu	X. Li LX200702 (GXF)	Napo, Guangxi, China	MW602132	MW602102
*Paraphlomishsiwenii* Y.P. Chen & Xiong Li 1	W.H. Wu et al. DD426 (KUN)	Jingxi, Guangxi, China	OP605346	OP609841
*Paraphlomishsiwenii* Y.P. Chen & Xiong Li 2	W.H. Wu et al. DD426 (KUN)	Jingxi, Guangxi, China	OP605347	OP609842
*Paraphlomisintermedia* C.Y. Wu & H.W. Li	X. Zhong et al. ZX16823 (CSH)	Suichang, Zhejiang, China	MW602135	MW602105
Paraphlomisjavanica(Blume)Prainvar.javanica 1	Y.P. Chen s.n. (KUN)	Kunming, Yunnan, China	MW602121	MW602088
Paraphlomisjavanica(Blume)Prainvar.javanica 2	L.B. Jia et al. JLB0029 (KUN)	Maguan, Yunnan, China	MW602143	MW602113
Paraphlomisjavanicavar.pteropoda D. Fang & K.J. Yan	X. Li 2020090501 (GXF)	Jingxi, Guangxi, China	MW602140	MW602110
*Paraphlomisjiangyongensis* X.L. Yu & A. Liu 1	A. Liu et al. LK1104 (CSFI)	Jiangyong, Hunan, China	MW602128	MW602095
*Paraphlomisjiangyongensis* X.L. Yu & A. Liu 2	A. Liu et al. LK1104 (CSFI)	Jiangyong, Hunan, China	MW602129	MW602096
*Paraphlomiskwangtungensis* C.Y. Wu & H.W. Li	Q. Fan et al. 19738 (SYS)	Qujiang, Guangdong, China	PP713070	PP706067
*Paraphlomislanceolata* Hand.-Mazz. 1	C.Z. Huang s.n. (KUN)	Guidong, Hunan, China	MW602145	MW602115
*Paraphlomislanceolata* Hand.-Mazz. 2	A. Liu et al. LK0825 (CSFI)	Ningyuan, Hunan, China	MW602146	MW602116
*Paraphlomislancidentata* Y.Z. Sun	X. Zhong et al. ZX16824 (CSH)	Suichang, Zhejiang, China	MW602136	MW602106
*Paraphlomislongicalyx* Y.P. Chen & C.L. Xiang	Y.P. Chen et al. EM583 (KUN)	Huanjiang, Guangxi, China	OK104771	OK104774
*Paraphlomismembranacea* C.Y. Wu & H.W. Li	M.S. Nuraliev 1057 (MW)	Thanh Son, Phu Tho, Vietnam	/	MW602100
*Paraphlomisnana* Y.P. Chen, C. Xiong & C.L. Xiang 1	C. Xiong XC21097 (KUN)	Chengkou, Chongqing, China	OM836062	OM884451
*Paraphlomisnana* Y.P. Chen, C. Xiong & C.L. Xiang 2	C. Xiong & H.L. Zhou XC21126 (KUN)	Wushan, Chongqing, China	OM836063	OM884452
*Paraphlomisoctopus* Q. Fan, Y.P. Chen & Ying Liu 1	Y.P. Chen & Y. Zhao EM1391 (KUN)	Huaiji, Guangdong, China	MW602126	MW602093
*Paraphlomisoctopus* Q. Fan, Y.P. Chen & Ying Liu 2	Q. Fan et al. 19752 (SYS)	Fengkai, Guangdong, China	PP713071	PP706068
*Paraphlomisoctopus* Q. Fan, Y.P. Chen & Ying Liu 3	Q. Fan et al. 19760 (SYS)	Pingle, Guangxi, China	PP713072	PP706069
*Paraphlomispagantha* Dunn 1	L.X. Yuan et al. s.n. (KUN)	Qionghai, Hainan, China	OP605345	OP609840
*Paraphlomispagantha* Dunn 2	X.Y. Jiang et al. HN001 (SYS)	Wenchang, Hainan, China	PP713073	PP706070
*Paraphlomispatentisetulosa* C.Y. Wu	C.L. Su et al. XY015 (KUN)	Xinyi, Guangdong, China	OQ627455	OQ628081
*Paraphlomispaucisetosa* C.Y. Wu 1	X.X. Zhu s.n. (KUN)	Malipo, Yunnan, China	MW602125	MW602092
*Paraphlomispaucisetosa* C.Y. Wu 2	X. Li LX200704 (GXF)	Napo, Guangxi, China	MW602133	MW602103
***Paraphlomisqingyuanensis* 1**	**Q. Fan et al. QYK-HH-1904 (SYS)**	**Yingde, Guangdong, China**	**PP897031**	**PP897952**
***Paraphlomisqingyuanensis* 2**	**Q. Fan et al. 20255 (SYS)**	**Yingde, Guangdong, China**	**PP897032**	**PP897953**
*Paraphlomisreflexa* C.Y. Wu & H.W. Li	Z.Z. Yang et al. s.n. (HIB)	Tongshan, Hubei, China	MW602122	MW602089
*Paraphlomisstrictiflora* J.C. Yuan, B. Chen & C.L. Xiang	B. Chen et al. CB05956 (CSH)	Yinjiang, Guizhou, China	/	OP609839
***Paraphlomissubcoriacea* 1**	**Q. Fan et al. QYK-CJ-1911 (SYS)**	**Yangshan, Guangdong, China**	**PP897033**	**PP897954**
***Paraphlomissubcoriacea* 2**	**Q. Fan et al. QYK-LB-1920 (SYS)**	**Yangshan, Guangdong, China**	**PP897034**	**PP897955**
*Paraphlomisyingdeensis* W.Y. Zhao, Y.Q. Li & Q. Fan	Q. Fan et al. 19013 (SYS)	Yingde, Guangdong, China	OP605348	OP609843
*Phlomisfruticosa* L.	Y. Tong s.n. (KUN)	Shanghai, China (cultivated)	MW602119	MW602086
Phlomoidesdentosavar.glabrescens (Danguy) C.L. Xiang & H. Peng	Y.P. Chen EM360 (KUN)	Beijing, China (cultivated)	MW602120	MW602087

## ﻿Appendix 2

**Table A2.** Populations information for PCA and Admixture analysis used in this study.

Population ID	Species	Voucher	GPS	Number of individual
P1	*Paraphlomisbaiwanensis*	Q. Fan 20251 (SYS); Hecang village, Baiwan town, Qingyuan city, Guangdong, China	24°15'09.40"N, 112°46'47.32"E	10
P2	*Paraphlomissubcoriacea*	Q. Fan 20257 (SYS); Xiatianxia, Lingbei town, Yangshan city, Guangdong, China	24°39'48.99"N, 112°45'41.73"E	15
P3	*Paraphlomissubcoriacea*	QYK-LB-1920 (SYS); Yanzidong, Lingbei town, Yangshan city, Guangdong, China	24°39'16.92"N, 112°46'59.95"E	6
P4	*Paraphlomissubcoriacea*	QYK-CJ-1911 (SYS); Baizhuling, Chengjia country, Yangshan city, Guangdong, China	24°39'﻿39.56"﻿N, 112°49'45.69"E	15
P5	*Paraphlomisqingyuanensis*	Q. Fan 20255 (SYS); Hegushi, Huanghua town, Yingde city, Guangdong, China	24°13'35.7"N, 112°56'08.97"E	14
P6	*Paraphlomisqingyuanensis*	QYK-HH-1882 (SYS); Huanghua park, Huanghua town, Yingde city, Guangdong, China	24°12'03.46"N, 112°54'10.05"E	15
P7	*Paraphlomisqingyuanensis*	QYK-HH-1904 (SYS); near Hegushi, Huanghua town, Yingde city, Guangdong, China	24°13'22.71"N, 112°56'05.71"E	10
